# Supplementary material for: Using SMART Magnetic Fluids and Gels for Prevention and Destruction of Bacterial Biofilms
Source: Microorganisms. 2023 Jun 7;11(6):1515. doi: 10.3390/microorganisms11061515 (PMC10305264; doi:10.3390/microorganisms11061515)
Supplement: Supplementary file 1 [file microorganisms-11-01515-s001.zip › microorganisms-2446098-supplementary.pdf]

Using SMART Magnetic Fluids and Gels for Prevention and Destruction of Bacterial Biofilms

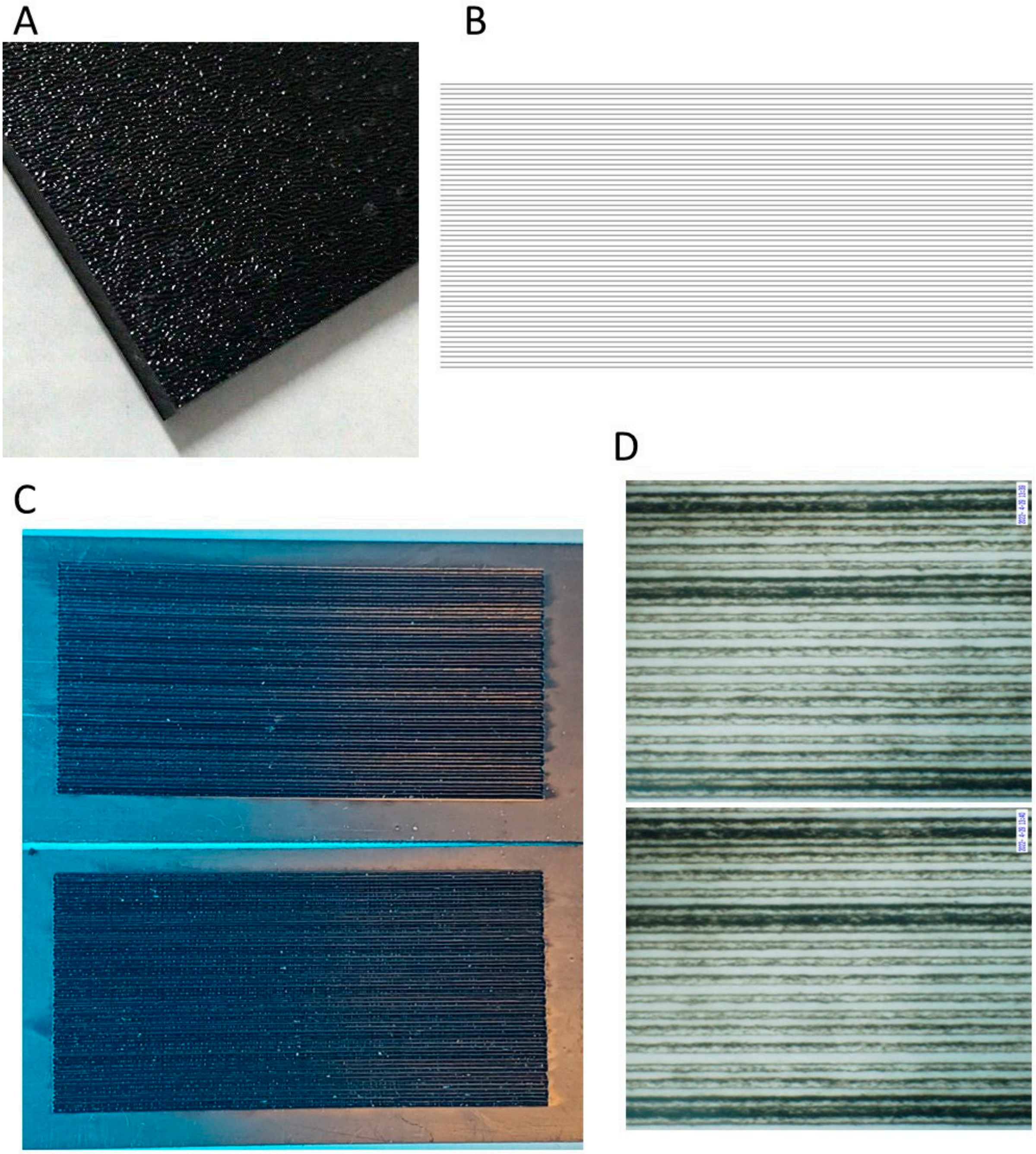

Fig. S1. Laser surface texturing of ABS plate. A) original single-side texture, B) grid file for texturing, C) engraved slides D) laser engraved surface.

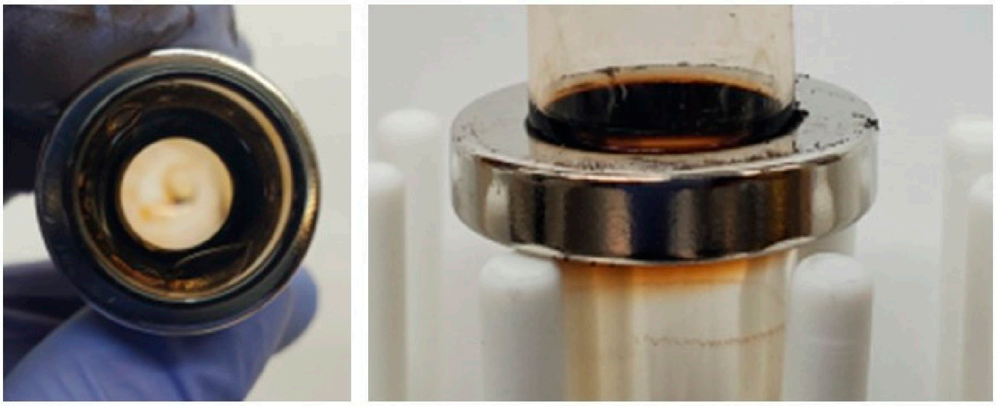

Fig. S2. Ring of FF inside the culture tube.

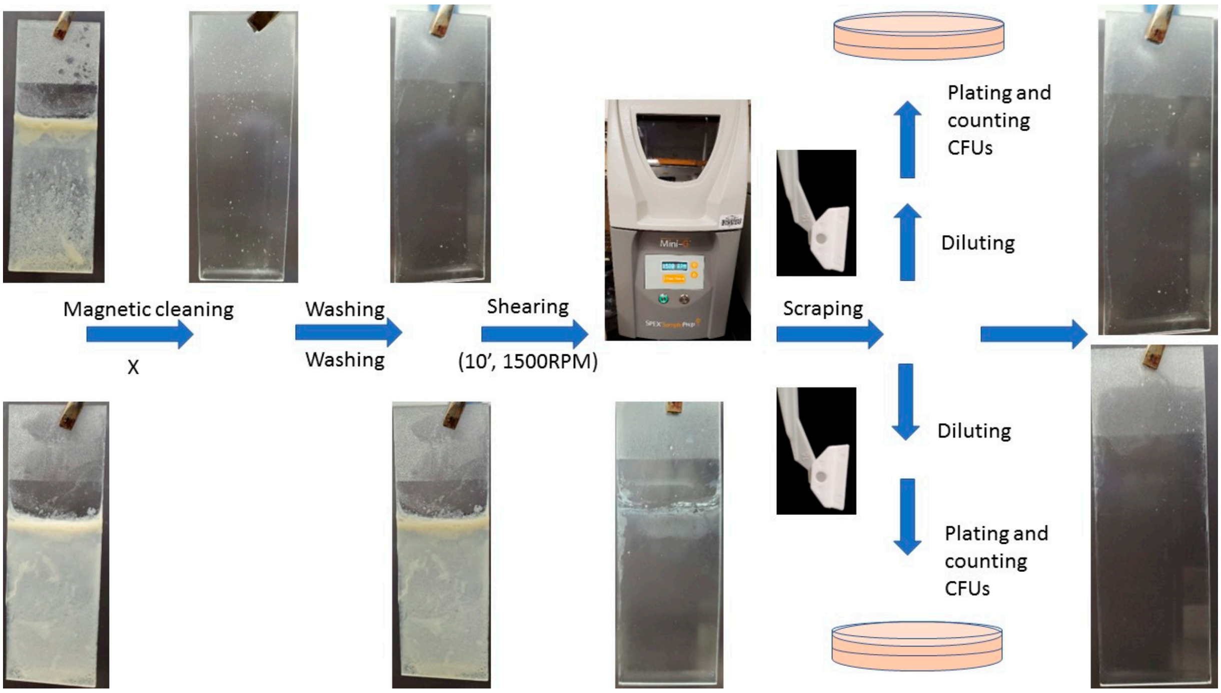

Fig. S3. Graphical representation of slide biofilm removal experiment with colony counting.

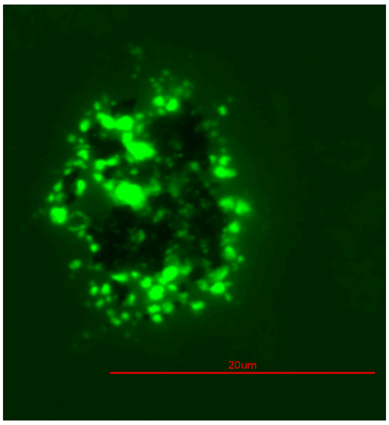

Fig. S4. Bacterial cells attached to the IO particles. Red bar scales represent 20 µm.

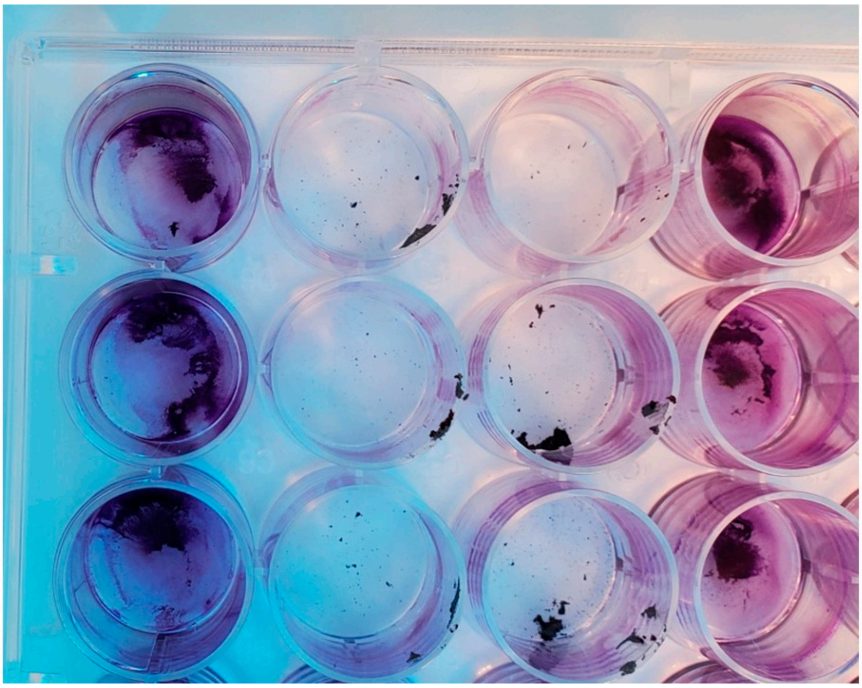

Fig. S5. Bacterial biofilm stained with CV attached to the bottom of a 24-well plate removed by SMARTFs. Rows: 1,4 not treated controls, 2-3 wells treated with MRFs and FG.
